# The degradation of proteins in pinniped skeletal muscle: viability of post-mortem tissue in physiological research

**DOI:** 10.1093/conphys/cov019

**Published:** 2015-05-25

**Authors:** Colby D. Moore, Andreas Fahlman, Daniel E. Crocker, Kathleen A. Robbins, Stephen J. Trumble

**Affiliations:** 1 Department of Biology, Baylor University, One Bear Place, Waco, TX 76706, USA; 2 Department of Life Sciences, Texas A&M University Corpus Christi, 6300 Ocean Drive, Corpus Christi, TX 78412, USA; 3 Department of Biology, Sonoma State University, 1801 East Cotati Avenue, Rohnert Park, CA 94928, USA

**Keywords:** Degradation, enzyme, myoglobin, pinniped

## Abstract

As marine divers, pinnipeds have a high capacity for exercise at depth while holding their breath. With finite access to oxygen, these species need to be capable of extended aerobic exercise and conservation of energy. Pinnipeds must deal with common physiological hurdles, such as hypoxia, exhaustion and acidosis, that are common to all exercising mammals. The physiological mechanisms in marine mammals used for managing oxygen and carbon dioxide have sparked much research, but access to animals and tissues is difficult and requires permits. Deceased animals that are either bycaught or stranded provide one potential source for tissues, but the validity of biochemical data from post-mortem samples has not been rigorously assessed. Tissues collected from stranded diving mammals may be a crucial source to add to our limited knowledge on the physiology of some of these animals and important to the conservation and management of these species. We aim to determine the reliability of biochemical assays derived from post-mortem tissue and to promote the immediate sampling of stranded animals for the purpose of physiological research. In this study, we mapped the temporal degradation of muscle enzymes from biopsied Northern elephant seals (*Mirounga angustirostris*) and highlight recommendations for storage protocols for the best preservation of tissue. We also compared the enzymatic activity of different muscle groups (pectoral and latissimus dorsi) in relation to locomotion and measured the effects of four freeze–thaw cycles on muscle tissue enzyme function. Results indicate that enzymatic activity fluctuates greatly, especially with varying storage temperature, storage time, species and muscle group being assayed. In contrast, proteins, such as myoglobin, remain relatively continuous in their increase at 4°C for 48 h. Stranded animals can be a valuable source of biochemical data, but enzyme assays should be used only with great caution in post-mortem tissues.

## Abstract (precis)

Post mortem tissue from stranded marine mammals is met with skepticism when utilized in biochemical assays. Degradation of muscle proteins and enzymes are not well defined. Given the protected nature and value of diving mammal samples, a quantitative analysis of degradation is valuable and presented here.

## Introduction

Muscle tissue samples collected *in vivo* have provided a vast amount of knowledge on the physiology, exercise performance and basic muscle structure of marine mammals (Kanatous *et al*., 1999, 2008; Dearolf *et al*., 2000; Watson *et al*., 2003; Trumble *et al*., 2010; Kielhorn *et al*., 2013; Velten *et al*., 2013). Research on marine mammal muscle tissue often focuses on aerobic and anaerobic properties and capacities of skeletal muscle deduced from biopsies (Lenfant *et al*., 1970; Castellini *et al*., 1981; Kooyman *et al*., 1981; Reed *et al*., 1994; Kanatous *et al*., 1999, 2002, 2008; Ponganis *et al*., 2002; Burns *et al*., 2005; Noren *et al*., 2005; Richmond *et al*., 2006; Clark *et al*., 2007; Spence-Bailey *et al*., 2007; Hindle *et al*., 2009; Prewitt *et al*., 2010; Shero *et al*., 2012; Moore *et al*., 2014). Obtaining marine mammal specimens for research purposes is often difficult. Small tissue biopsies from opportunistic captures during permitted research as well as subsistence hunts are used in physiology research (Reed *et al*., 1994; Kanatous *et al*., 1999; Polasek *et al*., 2006; Kanatous *et al*., 2008). It is less common for publications to include post-mortem tissue sampled from either bycaught or stranded specimens. Between 1999 and 2014, there have been approximately twice as many published papers using biopsy sampling in marine mammal research in comparison to post-mortem specimens (Kanatous *et al*., 2002, 2008; Ponganis *et al*., 2002; Burns *et al*., 2005; Noren *et al*., 2005; Richmond *et al*., 2006; Clark *et al*., 2007; Spence-Bailey *et al*., 2007; Hindle *et al*., 2009; Prewitt *et al*., 2010; Shero *et al*., 2012). The relative importance of post-mortem tissues, whether from euthanized stranded or bycaught animals, depends on whether intramuscular biochemical data collected from post-mortem species is feasible for tissue-based related physiological research. Previous studies using marine mammal tissues collected at post-mortem examination 24–72 h after collection and up to 30 h after death have yielded publishable results (Moore *et al*., 2009; Hoffman *et al*., 2013). Post-mortem samples collected within 6 h of death from stranded animals have been shown to complement data pertaining to physiological adaptations to depth and pressure (Watson *et al*., 2003, 2007; Polasek *et al*., 2006; Lestyk *et al*., 2009). After 6 h post-mortem, tissue integrity may be compromised due to decomposition or from sample handling or storage. There are a number of conditions that contribute or add to proteolysis, such as enzymatic activity (Geesink *et al*., 2006), temperature (Morita *et al*., 1996), disease state (Costelli *et al*., 2005), pH (Eijsink *et al*., 2005) and level of muscle atrophy (Kachaeva and Shenkman, 2012).

To determine the integrity of decomposed tissue and determine enzymatic activity and proteolysis over time with varying temperature regimens, skeletal muscle (latissimus dorsi; LD) was tested in a controlled laboratory setting, at a standard storage temperature (4°C), room temperature (21°C) and mammalian body temperature (37°C) for up to 48 h. These temperatures were chosen based on their common use in animal storage, post-mortem examination and transportation, respectively. Citrate synthase (CS), lactate dehydrogenase (LDH) and myoglobin (Mb) were chosen due to their common use in marine mammal literature as proxies for metabolic profiles (Castellini *et al*., 1981; Reed *et al*., 1994; Kanatous *et al*., 1999, 2008; Polasek *et al*., 2006). We hypothesized that the stability of enzymes would be greater in standard storage (4°C) when compared with higher temperatures (21 and 37°C) and that caution should be exercised when using skeletal muscle tissue from stranded individuals for enzymatic assays. In addition, we hypothesized that in order to maintain muscle integrity, immediate cold storage is necessary even with the risk of repeated freeze–thaw, because these conditions would be less detrimental than exposure to higher temperatures for even short periods of time. To our knowledge, this is the first study using marine mammal tissue to determine the degree and rate at which skeletal muscle becomes unusable for physiological investigations.

## Materials and methods

### Animals

Skeletal muscle biopsies were obtained from five live adult male Northern elephant seals (NES; *Mirounga angustirostris*; *n* = 5). In addition, California sea lions (CSL; *Zalophus californianus*; *n* = 2), one NES (*n* = 1) and one harbour seal (*Phoca vitulina*; *n* = 1) were sampled immediately post-mortem. For a terrestrial mammal comparison, biceps femoris skeletal muscle was extracted from a *Rattus rattus* (post-mortem, but not upon immediate death). All samples were immediately stored at −80°C for long-term storage, except field samples, which were placed into a liquid nitrogen dry shipper (Thermo Scientific) before they were transported overnight to −80°C. Northern elephant seal muscle samples were collected during muscle physiology research on Año Nuevo State Reserve (CA, USA) during beach haul-outs in 2013. Seals were anaesthetized with an intramuscular injection of Telazol, a teletamine/zolazepam hydrochloride, at a dose of ∼0.3 mg/kg (Crocker *et al*., 2012). Doses of ketamine and diazepam were also administered intravenously as needed to maintain immobilization (Fort Dodge Laboratories, Fort Dodge, IA, USA; Crocker *et al*., 2012). Latissimus dorsi muscle was accessed via incision after sterilization of the outer skin area (2 cm^2^ area). Biopsies (30–50 mg) were obtained in the mid-belly of the muscle at identical locations in all NES, using local Lidocaine^®^ (1 ml; Whitehouse Station, NJ, USA) and a 6 mm cannula (Depuy, Warsaw, IN, USA; Crocker *et al*., 2012). Samples were collected under National Marine Fisheries Service marine mammal permit #14636. All procedures were approved by Sonoma State University institutional animal care and use committee, and every precaution was taken to ensure that all biopsy samples were maintained in a sterile environment from sampling through assay. Post-mortem marine mammal samples were obtained from The Marine Mammal Center in Sausalito (CA, USA) under permit #932-1905-00/MA-009526.

### Assay protocols

Northern elephant seal skeletal muscle was thawed specifically for assay and immediately subjected to two temperatures (4 or 21°C) and four time intervals (3, 12, 24 and 48 h) during decomposition studies. Skeletal muscle was homogenized using a Bullet Blender (0.5 mm zirconium oxide beads; Next Advance, Averill Park, NY, USA) in Sigma CellLytic MT buffer (Sigma Aldrich).

Citrate synthase assays were performed on a Beckman Coulter DU 730 spectrophotometer according to the Sigma Aldrich protocol (CS0720). Briefly, and according to the Sigma Aldrich protocol (CS0720), the activity level (in micromoles per minute per gram) was determined at a wavelength of 412 nm by combining the protein sample, assay buffer, acetyl CoA solution, dithiobis-nitrobenzoic acid (DTNB) solution and oxaloacetic acid (OAA) solution. The reaction of acetyl CoA and OAA to citrate followed the colorimetric reaction of DTNB to TNB, forming a yellow colour. The reaction was followed for 1.5 min to measure the baseline. The OAA was added, and after another 1.5 min the total activity was measured. Results were based on the change in absorbance at 412 nm over 1 min and the extinction coefficient of TNB, as outlined in the Sigma Aldrich protocol (CS0720).

The effects of four freeze–thaw cycles on skeletal muscle enzyme concentration were examined in the rat muscle only. Muscle was maintained at −80°C during the freeze event and thawed repeatedly. In the course of one freeze–thaw event, muscle was frozen at −80°C, thawed completely (completed in a matter of minutes in the small tissue samples), homogenized and used in the citrate synthase enzymatic assay. Each freeze–thaw cycle measurement was made 24 h apart; thus, the muscle was fully refrozen before the subsequent measurement was made.

Lactate dehydrogenase assays were performed according to the Sigma Aldrich protocol (MAK066) on a spectrophotometric multiwell plate reader (Beckman Coulter, DTX880). The quantification of LDH was based on the catalysis of the interconversion of pyruvate and lactate, which reduced NAD to NADH and was detected at 450 nm. Briefly, and according to Sigma Aldrich protocol (MAK066), protein samples were mixed with LDH assay buffer and a master reaction mix containing buffer and LDH substrate. Samples were then rotated between incubation at 37°C and measurements every 5 min until activity surpassed the highest standard.

Myoglobin assays were completed using methodology modified by Kanatous *et al*. (1999) from Reynafarje (1963). Homogenates were diluted in phosphate buffer (0.4 m potassium phosphate at pH 6.6) and centrifuged at 28 000***g*** for 50 min. The supernatant was bubbled with carbon monoxide for 3 min before being measured for spectrophotometric absorbance. Absorbance was measured at two wavelengths (538 and 568 nm), and Mb concentration was calculated in milligrams per gram of wet muscle mass.

### Statistical analysis

Group differences were assessed using ANOVA followed by Tukey–Kramer HSD test. Results were analysed with statistical significance at *P* ≤ 0.05 α level. Results are presented as means ± SEM.

## Results

**Figure 1:**
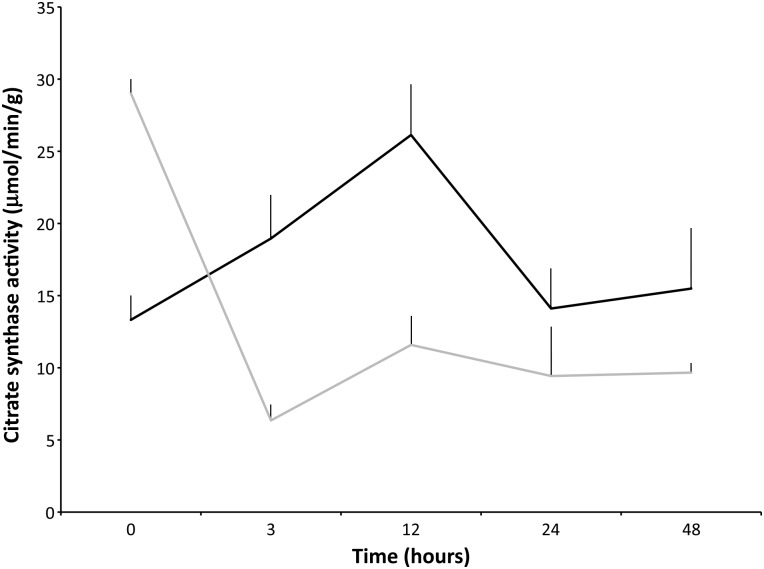
Citrate synthase activity level (in micromoles per minute per gram; mean values ± SEM) in the longissimus dorsi muscle of four Northern elephant seal adult males. Measurements were made over 48 h at two different temperatures [black represents 4°C (individuals ES13-M3 and ES13-M12) and grey 21°C (individuals ES13-M13 and ES13-M4)], indicating the greater stability of the enzyme at 4 vs. 21°C in biopsied muscle tissue. ES represents “elephant seal”, 13 is the year of collection and “M” is male.

**Figure 2:**
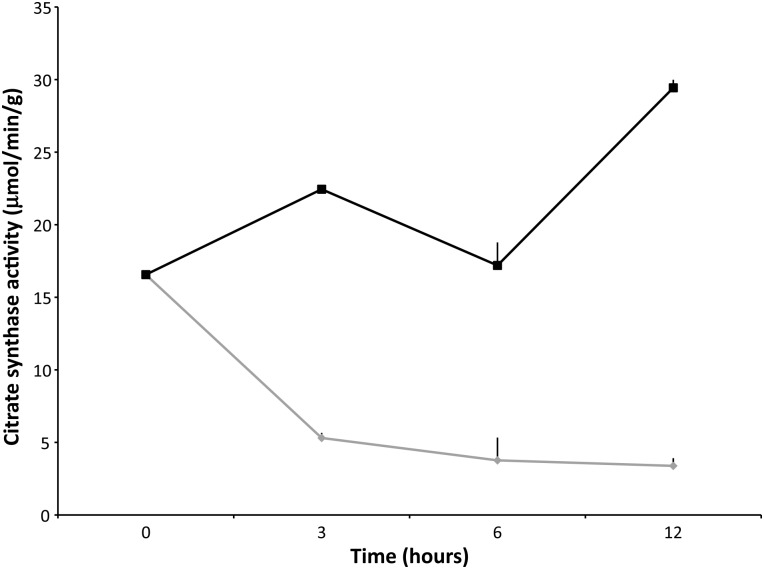
Average citrate synthase activity level (in micromoles per minute per gram; mean values ± SEM) in a rat locomotory muscle measured in triplicate (*n* = 1). Measurements were made over 12 h at two temperatures (black represents 4°C and grey 37°C), indicating the greater stability of the enzyme at 4 than at 37°C.

Overall, there was an increase in citrate synthase activity between samples maintained at 4 vs. 21°C, from 3 to 48 h (Fig. 1; ANOVA, *P* < 0.05). The CS activity level was measured for four adult male NES (Fig. 1 and Table 1). Measurements were made over 48 h at five time points (0, 3, 12, 24 and 48 h) at 4 and 21°C (Fig. 1). At each time point after 0 h, the 4°C group had elevated enzymatic activity compared with the 21°C group (Fig. 1; Tukey–Kramer HSD, *P* < 0.05). For biopsies maintained at 4°C, CS enzymatic activity increased over time up to 12 h (26.1 ± 3.5 μmol/min/g, Fig. 1; Tukey–Kramer HSD, *P* < 0.05). The percentage change in CS activity level (4°C) over four sampling time frames (0–3, 3–12, 12–24 and 24–48 h; Table 2) fluctuated from a 42.3% increase to a 46% decrease, demonstrating the instability of this enzyme over time. The percentage change in CS activity level at 21°C decreased, with the largest negative percentage change from 0 to 3 h (−78.1%) in comparison to 21°C from 3 to 12 h (82.4%). The CS activity level was also measured in a rat locomotory muscle (biceps femoris; Fig. 2). The CS activity in the rat muscle was elevated at 4°C (range from 16.0 ± 0.0 to 29.4 ± 0.5 μmol/min/g) when compared with 37°C (range from 3.4 16 ± 0.5 to 16 ± 0 μmol/min/g; Fig. 2; Student’s unpaired *t*-test, *P* < 0.05). Therefore, the CS activity level was greater in both NES and rat tissues maintained at 4°C. For the 37°C rat muscle, CS activity was significantly decreased after time 0 h (Fig. 2; Tukey–Kramer HSD, *P* < 0.05). The effects of four freeze–thaw cycles on the degradation of rat skeletal muscle showed no statistical difference in CS activity when thawed at 24 h. Therefore, the CS activity level was relatively stable when the muscle was maintained at −80°C, even after four consecutive freeze–thaw cycles (Table 3; Tukey–Kramer HSD, *P* > 0.05).

**Table 1:** Citrate synthase (in micromoles per minute per gram; mean values ± SEM) and lactate dehydrogenase (in milliunits per millilitre; mean values ± SEM) for five adult Northern elephant seals over 24 and 48 h at two different temperatures: 4°C [ES13-M3 (CS), ES13-M2 (LDH) and ES13-M12] and 21°C (ES13-M4 and ES13-M13)

Time	CS at 4°C	CS at 21°C	LDH at 4°C	LDH at 21°C
0 h	13.3 ± 1.6	29.0 ± 1.0	48.9 ± 7.8	39.6 ± 4.5
3 h	18.9 ± 3.0	6.3 ± 1.1	127.6 ± 44.7	54.1 ± 4.0
12 h	26.1 ± 3.5	11.6 ± 2.0	89.4 ± 9.5	50.6 ± 35.5
24 h	14.1 ± 2.8	9.4 ± 3.4	28.7 ± 12.6	30.4 ± 21.7
48 h	15.5 ± 4.2	9.7 ± 0.7	—	—

Abbreviations: CS, citrate synthase; LDH, lactate dehydrogenase. *n* = 5.

**Table 2:** Percentage change in citrate synthase and lactate dehydrogenase activity over four time frames at two temperatures (4 and 21°C)

Time frame (h)	CS 4°C (%)	CS 21°C (%)	LDH 4°C (%)	LDH 21°C (%)
0–3	42.3	−78.1	160.9	36.7
3–12	37.8	82.4	−29.9	−6.5
12–24	−46.0	−18.6	−67.9	−39.8
24–48	9.8	2.5	—	—

Abbreviations: CS, citrate synthase; LDH, lactate dehydrogenase. *n* = 5.

**Table 3:** Citrate synthase activity level (in micromoles per minute per gram; mean values ± SEM) for a rat after three freeze–thaw cycles

Freeze times	CS activity (μmol/min/g)
Baseline	14.3 ± 0.2
Freeze cycle 1	13.9 ± 0.5
Freeze cycle 2	13.9 ± 0.6
Freeze cycle 3	14.3 ± 1.5

Abbreviation: CS, citrate synthase. *n* = 1.

**Figure 3:**
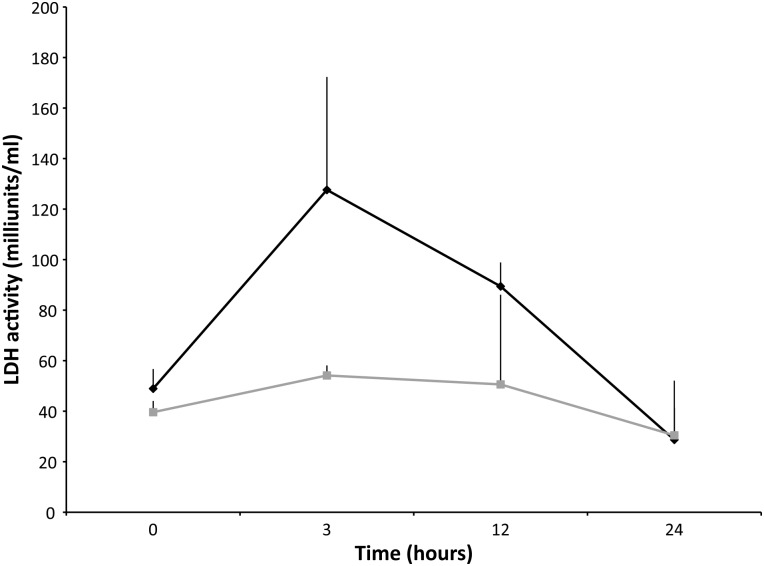
Lactate dehydrogenase activity level (in miliunits per millilitre; mean values ± SEM) in the longissimus dorsi muscle of four Northern elephant seal adult males. Measurements were made over 24 h at two different temperatures (black represents 4°C and grey 21°C), indicating the greater stability of the enzyme at 4 vs. 21°C in biopsied muscle tissue.

The mean LDH activity level (in milliunits per millilitre of extract) measured at times 0, 3, 12 and 24 h in four adult male NES (Fig. 3 and Table 1) revealed a similar pattern to CS, in that greater enzyme activity was evident at 4 than at 21°C (Fig. 3). Overall, there was a statistical difference between animals maintained at 4 vs. 21°C, from time 0 to 24 h (ANOVA, *P* < 0.05). Therefore, the LDH activity level was higher in tissues maintained at 4°C over a 24 h period.

**Figure 4:**
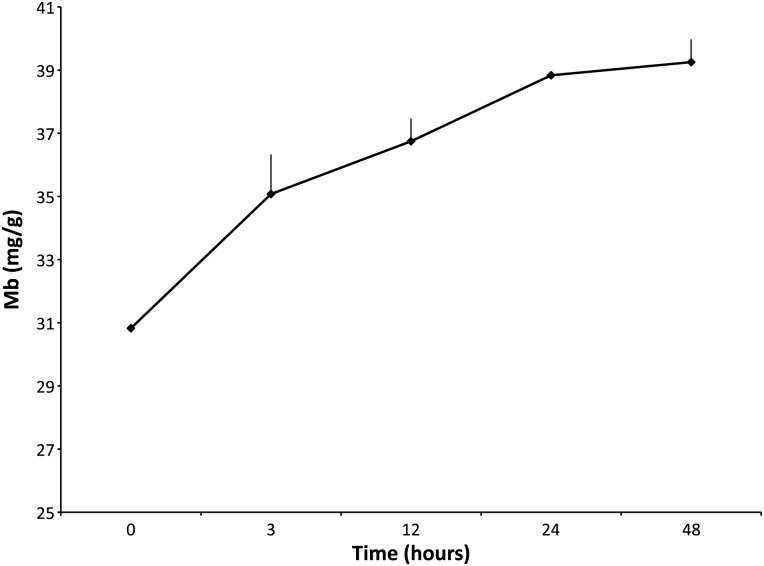
Northern elephant seal (individual ES3289) measured over 48 h at 4°C for degradation of myoglobin (Mb; in milligrams per gram; mean values ± SEM). The figure indicates the increase of myoglobin over time when maintained at 4°C.

The change in Mb concentration as a function of time during decomposition was measured in one elephant seal (ES3289) over 48 h at 4°C (Fig. 4 and Table 4). Myoglobin values increased significantly from time 0 to 48 h (Fig. 4 and Table 4; Tukey–Kramer HSD, *P* < 0.05). The average percentage increase in Mb over 48 h held at 4°C was 27.3% (Fig. 4 and Table 4).

**Table 4:** Northern elephant seal (individual ES3289) myoglobin concentration (in milligrams per gram; mean values ± SEM) over 48 h, maintained at 4°C

Time (h)	Myoglobin (mg/g)	Rate of change over 48 h (%)
0	30.8 ± 0	—
3	35.1 ± 1.2	14.0
12	36.7 ± 0.7	4.6
24	38.8 ± 0	5.7
48	39.2 ± 0.7	1.0

*n* = 1.

**Figure 5:**
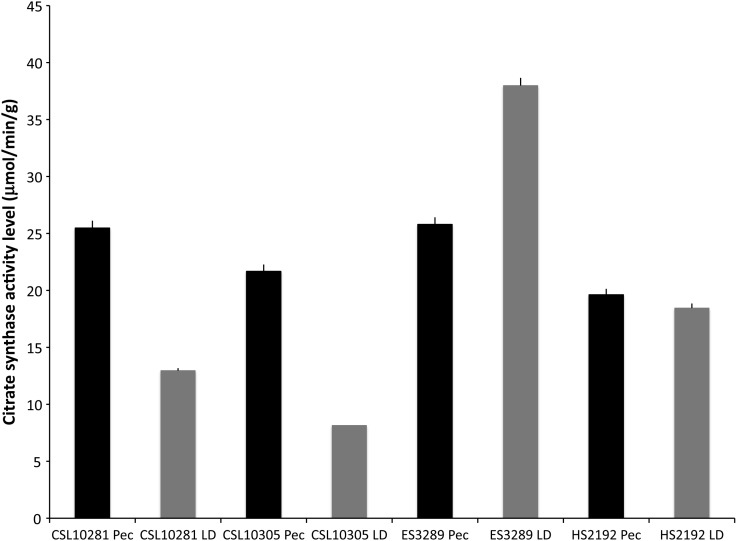
Citrate synthase activity level (in micromoles per minute per gram; mean values + SEM) in the pectoral and longissimus dorsi muscle of four different marine mammals (indicated by the different alphanumerical codes), indicating the general trend for higher values in pectoral muscle of those animals that predominately use the pectoral muscle for locomotion (California sea lions).

Citrate synthase activity was measured in both the pectoralis major and LD muscle in four post-mortem marine mammals (with different time of death to sampling intervals) stored at −20°C to compare the relative activity level between the two muscles (Fig. 5). Both CSLs (CSL10281 and CSL10305) demonstrated significantly increased levels of CS activity in pectoral muscle compared with LD muscle (ANOVA, *P* < 0.05). The elephant seal (ES3289) showed elevated levels of CS in the LD muscle (ANOVA, *P* < 0.05). Citrate synthase enzymatic activity in the harbour seal (HS2192) showed no significant difference in pectoral muscle vs. LD (ANOVA, *P* > 0.05). Therefore, for three of the four marine mammals the primary locomotory muscle used (pectoralis in CSL and LD in NES) had elevated CS activity (Fig. 5).

## Discussion

Enzymatic assays, such as for CS and LDH, may not provide reliable results, because the enzymes tend to be relatively unstable over 24 h at both room (21°C) and refrigerator temperature (4°C). Myoglobin concentrations were more constant than either CS or LDH enzymes and showed a general increasing trend (1–14%: Table 4). In addition, and unexpectedly, rat skeletal muscle enzyme (CS) concentrations were not significantly different when assessed between freeze–thaw intervals. These data indicate the necessity for immediate (on-location) storage at temperatures below 0°C for skeletal muscle tissue from both live and recently deceased animals.

Enzyme stability is related to a number of factors, including pH, temperature and oxidative stress (Eijsink *et al*., 2005). Between time 0 and 24 h, tissues maintained at 4°C (ES13-M3, ES13-M12) demonstrated a 6% increase in CS activity, whereas the muscle tissues maintained at 21°C (ES13-M13, ES13-M4) showed a degradation of 68% (from 29.0 ± 1.0 to 9.4 ± 3.4“ μmol/min/g) over 24 h.

When rat tissue was exposed to a high temperature (37°C), the CS activity level decreased significantly compared with 4°C (Fig. 2), lending support to our hypothesis that temperature may cause a pronounced degradation of the CS enzyme in NES. Our findings are in agreement with previous findings that CS activity decreases at temperatures above 40°C (Zhi *et al*., 1991) and inactivation of the enzyme is reached at 43°C (Jakob *et al*., 1995). Thus, CS is very sensitive to thermal stress (Jakob *et al*., 1995) and, depending on the environmental conditions at time of death and the storage temperature, CS for aerobic determination stored in these conditions should not be used. Citrate synthase activity varied among the NES skeletal muscle samples at time 0 h, therefore, regardless of time and temperature (Fig. 1). This correlates well with previous research indicating that muscle tissue post-mortem can be variable even within the same muscle group of the same animal (Bendall, 1973). Given the large amount of variability among NES adult males (Fig. 1), the results suggest that CS may not be a reliable enzyme as a proxy for aerobic metabolism between animals, even in fresh biopsy samples. Although freezing can affect the muscle tissue in various ways, including formation of ice crystals, dehydration and denaturation of proteins (Jeong *et al*., 2011), the results from the present study suggest that freezing is the best method of preservation. Evidence for frozen storage was demonstrated by rat skeletal muscle repeatedly frozen four times, resulting in no difference detected in mean CS activity among freeze–thaw cycles (Table 3). However, some caution should be taken with successive freeze–thaw cycles, because variability (SEM) appeared to increase with increasing numbers of freeze events.

Lactate dehydrogenase is also sensitive to thermal conditions (Adler and Lee, 1999), with a 15% decline in activity when stored at 4°C for 4 days (Jacobs *et al*., 1986) and 5% at 4°C after 24 h (Wagner *et al*., 1992). This study showed that for NES skeletal muscle subjected to both 4 and 21°C for 24 h, LDH activity decreased by 41.3 and 23.2%, respectively (Fig. 3 and Table 1). Although LDH was less variable at 4 than at 21°C (Fig. 3), the increased variability of LDH at both temperatures indicates that enzyme data from muscle stored at temperatures above freezing should be analysed with caution. In addition, and much like CS, the variability among NES adult males may indicate that LDH used as a proxy for metabolism may not be reliable.

In veterinary clinical cases, as with the response to stranding, tissue biopsy samples are often collected and stored at different temperatures for shipping and/or long-term storage (Stanley *et al*., 2009). The repeated freeze–thaw of samples is unavoidable and may impact the integrity of samples. It has been reported from studies in the food industry that the freeze–thaw process can be detrimental to the overall quality of the tissue sample, due primarily to increased lipid oxidation and tissue aesthetics with regard to its exposure to temperature alteration (Tang *et al*., 2006; Jeong *et al*., 2011). Our data show that when assessing the CS enzyme, valuable data can be obtained even in the event that samples are thawed. No significant change in CS enzyme activity occurs with successive freeze–thawing cycles. Furthermore, tissue samples should be placed immediately into −80°C, primarily because muscle storage over time and elevated temperature can change enzymatic activity in as little as 3 h even at refrigerator temperature (4°C; Table 1).

In the present study, we observed that Mb held at 4°C for up to 48 h increased in a relatively constant manner. The Mb concentration increased maximally by 8.4 mg/g (27%, time 0–48 h), compared with LDH, which fluctuated by 78.7 miliunits/ml (161%) at 4°C (Tables 1 and 4). In addition, the variability (SEM) associated with the Mb averages (0–1.2 mg/g) was generally lower than SEM values for either CS or LDH (1.6–4.2 μmol/min/g and 7.8–44.7 milliunits/ml, respectively; Tables 1 and 4). While these changes in Mb would alter the precision of muscle oxygen store measurements, they suggest reasonable utility of post-mortem samples for defining species differences, especially for species that are hard to sample.

During this study, we also detected that enzymatic activity varied between different locomotory skeletal muscle groups within the same animal (Fig. 5). To determine species specificity and differences in CS activity levels between skeletal muscle groups, CS activity was measured in the pectoralis major and LD muscle in three individual pinnipeds representing different primary locomotion approaches; NES (hindflipper), CSL (foreflipper) and harbour seal (hind-/foreflipper). The greatest level of CS activity was found in the NES LD, the primary locomotory muscle of this deep-diving phocid seal (Le Boeuf *et al*., 2000; Kuhn *et al*., 2009; Robinson *et al*., 2012; Fig. 5). For the CSL, a relatively shallow-diving Otariid species (Feldkamp *et al*., 1989; Weise *et al*., 2006), the highest CS activity was found in the pectoralis major when compared with the CSL LD (Fig. 5). No difference was found between CS activity in pectoralis major and LD muscles for the comparatively intermediate-diving phocid, the harbour seal (Fig. 5). This may be indicative of the relative equal reliance on fore- and hindflippers of the harbour seal vs. the elephant seal or the relative lack of utilization of pectoralis major compared with the CSL. These CS activity data provide indirect evidence designating which skeletal muscle group is the primary locomotory muscle in these marine mammal species (Feldkamp, 1987). We suggest that CS enzymatic activity levels can provide data on the relative importance of individual muscles to locomotion. Although enzymatic data may not provide a reliable comparison across species or even within species, we suggest that comparing between muscle groups within the same individual provides valuable data.

The aim of this study was to quantify enzyme degradation in marine mammal tissues collected post-mortem. We suggest that our findings substantiate the expedited use of post-mortem tissue and provide evidence that tissue is of greater value when refrigerated or frozen immediately following removal from an animal. When using enzyme assays to determine aerobic capacity, one may find that values have a large range, even among biopsies from the same species and age class, potentially indicating the fluctuating nature of these enzymes normally. We suggest that data collected from stranded (post-mortem) marine mammals can be valuable and we promote the quantification of stable proteins, such as Mb, if tissues have been maintained at 4°C. In addition, we suggest the immediate storage of tissues at temperatures below freezing (liquid nitrogen) and, in the event of a freeze–thaw cycle, muscle will maintain more integrity when refrozen compared with leaving tissues at refrigerator or room temperature for extended periods of time. It is important to note the difficulty in obtaining marine mammal samples and the scientific value that tissue from stranded individuals can provide to conservation efforts. Thus, it is imperative that researchers maintain the integrity of valuable samples for physiological research.

## Funding

This work was supported by the Office of Naval Research [grant number N00014-12-1-0187].

## References

[COV019C1] Adler M Lee G (1999) Stability and surface activity of lactate dehydrogenase in spray-dried trehalose . J Pharm Sci 88: 199–208. 9950639 10.1021/js980321x

[COV019C2] Bendall JR (1973) Post mortem changes in muscle . In Bourne GH, ed., The Structure and Function of Muscle. Academic Press , New York, pp 244–309.

[COV019C4] Burns JM Costa DP Frost K Harvey JT (2005) Development of body oxygen stores in harbor seals: effects of age, mass, and body composition . Physiol Biochem Zool 78: 1057–1068. 16228944 10.1086/432922

[COV019C5] Castellini MA Somero GN Kooyman GL (1981) Glycolytic enzyme activities in tissues of marine and terrestrial mammals . Physiol Zool 54: 242–252.

[COV019C6] Clark AC Burns JM Schreer JF Hammill MO (2007) A longitudinal and cross-sectional analysis of total body oxygen store development in nursing harbor seals (*Phoca vitulina*) . J Comp Physiol B 177: 217–227. 17089167 10.1007/s00360-006-0123-6

[COV019C7] Costelli P Reffo P Penna F Autelli R Bonelli G Baccino FM (2005) Ca^2+^-dependent proteolysis in muscle wasting . Int J Biochem Cell Biol 37: 2134–2146. 15893952 10.1016/j.biocel.2005.03.010

[COV019C100] Crocker DE Houser DS Webb PM (2012) Impact of body reserves on energy expenditure, water flux, and mating success in breeding male northern elephant seals . Physiol Biochem Zool 85: 11–20. 22237285 10.1086/663634

[COV019C8] Dearolf JL McLellan WA Dillaman RM Frierson D Jr Pabst DA (2000) Precocial development of axial locomotor muscle in bottlenose dolphins (*Tursiops truncatus*) . J Morphol 244: 203–215. 10815003 10.1002/(SICI)1097-4687(200006)244:3<203::AID-JMOR5>3.0.CO;2-V

[COV019C9] Eijsink VGH Gaseidnes S Borchert TV Van der Burg B (2005) Directed evolution of enzyme stability . Biomol Eng 22: 1–3. 15857780 10.1016/j.bioeng.2004.12.003

[COV019C10] Feldkamp SD (1987) Swimming in the California sea lion: morphometrics, drag and energetics . J Exp Biol 131: 117–135. 3694112 10.1242/jeb.131.1.117

[COV019C11] Feldkamp SD DeLong RL Antonelis GA (1989) Diving patterns of California sea lions, *Zalophus californianus* . Can J Zool 67: 872–883.

[COV019C12] Geesink GH Kuchay S Chishti AH Koohmaraie M (2006) μ-Calpain is essential for postmortem proteolysis of muscle proteins . J Anim Sci 84: 2834–2840. 16971586 10.2527/jas.2006-122

[COV019C13] Hindle AG Horning M Mellish JA Lawler JM (2009) Diving into old age: muscular senescence in a large-bodied, long-lived mammal, the Weddell seal (*Leptonychotes weddellii*) . J Exp Biol 212: 790–796. 19251994 10.1242/jeb.025387

[COV019C14] Hoffman JI Thorne MAS Trathan PN Forcada J (2013) Transcriptome of the dead: characterization of immune genes and marker development from necropsy samples in a free-ranging marine mammal . BMC Genomics 14: 1–14. 23347513 10.1186/1471-2164-14-52 PMC3563519

[COV019C15] Jacobs E Hissin PJ Propper W Mayer L Sarkozi L (1986) Stability of lactate dehydrogenase at different storage temperatures . Clin Biochem 19: 183–188. 3731436 10.1016/s0009-9120(86)80021-2

[COV019C16] Jakob U Lilie H Meyer I Buchner J (1995) Transient interaction of Hsp90 with early unfolding intermediates of citrate synthase . J Biol Chem 270: 7288–7294. 7706269 10.1074/jbc.270.13.7288

[COV019C17] Jeong J-Y Kim G-D Yang H-S Joo S-T (2011) Effect of freeze–thaw cycles on physicochemical properties and color stability of beef *semimembranosus* muscle . Food Res Int 44: 3222–3228.

[COV019C18] Kachaeva EV Shenkman BS (2012) Various jobs of proteolytic enzymes in skeletal muscle during unloading: facts and speculations . J Biomed Biotechnol 2012: 1–15. doi:10.1155/2012/493618. 22496611 10.1155/2012/493618 PMC3303694

[COV019C19] Kanatous SB DiMichele LV Cowan DF Davis RW (1999) High aerobic capacities in the skeletal muscle of pinnipeds: adaptations to diving hypoxia . J Appl Physiol 86: 1247–1256. 10194210 10.1152/jappl.1999.86.4.1247

[COV019C20] Kanatous SB Davis RW Watson R Polasek L Williams TM Mathieu-Costello O (2002) Aerobic capacities in the skeletal muscles of Weddell seals: key to longer dive durations? J Exp Biol 205: 3601–3608. 12409486 10.1242/jeb.205.23.3601

[COV019C200] Kanatous SB Hawke TJ Trumble SJ Pearson LE Watson RR Garry DJ Williams TM Davis RW (2008) The ontogeny of aerobic and diving capacity in the skeletal muscles of Weddell seals . J Exp Biol 211: 2559–2565. 18689409 10.1242/jeb.018119

[COV019C21] Kielhorn CE Dillman RM Kinsey ST McLellan WA Gay DM Dearolf JL Pabst DA (2013) Locomotor muscle profile of a deep (*Kogia breviceps*) versus shallow (*Tursiops truncatus*) diving cetacean . J Morphol 274: 663–675. 23355398 10.1002/jmor.20124

[COV019C23] Kooyman GL Castellini MA Davis RW (1981) Physiology of diving in marine mammals . Annu Rev Physiol 43: 343–356. 7011189 10.1146/annurev.ph.43.030181.002015

[COV019C24] Kuhn CE Crocker DE Tremblay Y Costa DP (2009) Time to eat: measurements of feeding behaviour in a large marine predator, the northern elephant seal *Mirounga angustirostris* . J Anim Ecol 78: 513–523. 19040681 10.1111/j.1365-2656.2008.01509.x

[COV019C25] Le Boeuf BJ Crocker DE Costa DP Blackwell SB Webb PM Houser DS (2000) Foraging ecology of northern elephant seals . Ecol Monogr 70: 353–382.

[COV019C26] Lecker SH Solomon V Mitch WE Goldberg AL (1999) Muscle protein breakdown and the critical role of the ubiquitin-proteasome pathway in normal and disease states . J Nutr 129: 227S–237S. 9915905 10.1093/jn/129.1.227S

[COV019C27] Lenfant C Johansen K Torrance JD (1970) Gas transport and oxygen storage capacity in some pinnipeds and the sea otter . Resp Physiol 9: 277–286. 10.1016/0034-5687(70)90076-9 5445188

[COV019C28] Lestyk KC Folkow LP Blix AS Hammill MO Burns JM (2009) Development of myoglobin concentration and acid buffering capacity in harp (*Pagophilus groenlandicus*) and hooded (*Cystophora cristata*) seals from birth to maturity . J Comp Physiol B 179: 985–996. 19565249 10.1007/s00360-009-0378-9

[COV019C29] Moore C Crocker DE Fahlman A Moore M Willoughby DS Robbins K Kanatous SB Trumble SJ (2014) Ontogenetic changes in skeletal muscle fiber type, fiber diameter and myoglobin concentration in the Northern elephant seal (*Mirounga angustirostris*) . Front Physiol 5: 217 doi:10.3389/fphys.2014.00217. 24959151 10.3389/fphys.2014.00217 PMC4050301

[COV019C30] Moore MJ Bogomolni AL Dennison SE Early G Garner MM Hayward A Lentell BJ Rotstein DS (2009) Gas bubbles in seals, dolphins, and porpoises entangled and drowned at depth in gillnets . Vet Pathol 46: 536–547. 19176498 10.1354/vp.08-VP-0065-M-FL

[COV019C31] Morita S Tsujinaka T Yano M Ebisui C Morimoto T Fujita J Ogawa A Taniguchi M Shiozaki H Monden M (1996) Temperature-dependent enhancement of proteolysis in C2C12 myotubes in association with the activation of 26S proteasome . Biochem Biophys Res Commun 228: 813–818. 8941359 10.1006/bbrc.1996.1737

[COV019C32] Noren SR Iverson SJ Boness DJ (2005) Development of the blood and muscle oxygen stores in gray seals (*Halichoerus grypus*): implications for juvenile diving capacity and the necessity of a terrestrial postweaning fast . Physiol Biochem Zool 78: 482–490. 15957103 10.1086/430228

[COV019C33] Polasek LK Dickson KA Davis RW (2006) Metabolic indicators in the skeletal muscles of harbor seals (*Phoca vitulina*) . Am J Physiol Regul Integr Comp Physiol 290: R1720–R1727. 16397095 10.1152/ajpregu.00080.2005

[COV019C34] Ponganis PJ Kreutzer U Stockard TK Lin PC Sailasuta N Tran TK Hurd R Jue T (2002) Blood flow and metabolic regulation in seal muscle during apnea . J Exp Biol 211: 3323–3332. 18840667 10.1242/jeb.018887

[COV019C35] Prewitt JS Freistroffer DV Schreer JF Hammill MO Burns JM (2010) Postnatal development of muscle biochemistry in nursing harbor seal (*Phoca vitulina*) pups: limitations to diving behavior? J Comp Physiol B 180: 757–766. 20140678 10.1007/s00360-010-0448-z

[COV019C36] Reed JZ Butler PJ Fedak MA (1994) The metabolic characteristics of the locomotory muscles of grey seals (*Halichoerus grypus*), harbor seals (*Phoca vitulina*) and Antarctic fur seals (*Arctocephalus gazella*) . J Exp Biol 194: 33–46. 7964404 10.1242/jeb.194.1.33

[COV019C37] Reynafarje B (1963) Method for the determination of myoglobin . J Lab Clin Med 61: 138–145. 13981912

[COV019C38] Richmond JP Burns JM Rea LD (2006) Ontogeny of total body oxygen stores and aerobic dive potential in Stellar sea lions (*Eumetopias jubatus*) . J Comp Physiol B 176: 535–545. 16514541 10.1007/s00360-006-0076-9

[COV019C39] Robinson PW Costa DP Crocker DE Gallo-Reynoso JP Champagne CD Fowler MA Goetsch C Goetz KT Hassrick JL Hückstädt LA (2012) Foraging behavior and success of a mesopelagic predator in the Northeast Pacific Ocean: insights from a data-rich species, the northern elephant seal . Plos ONE 7: e36728 doi:10.1371/journal.pone.0036728. 22615801 10.1371/journal.pone.0036728 PMC3352920

[COV019C40] Shero MR Andrews RD Lestyk KC Burns JM (2012) Development of the aerobic dive limit and muscular efficiency in northern fur seals (*Callorhinus ursinus*) . J Comp Physiol B 182: 425–436. 22001970 10.1007/s00360-011-0619-6

[COV019C41] Spence-Bailey LM Verrier D Arnould JP (2007) The physiological and behavioural development of diving in Australian fur seal (*Arctocephalus pusillus doriferus*) pups . J Comp Physiol B 177: 483–494. 17294194 10.1007/s00360-007-0146-7

[COV019C42] Stanley RL Maile C Piercy RJ (2009) Storage-associated artefact in equine muscle biopsy specimens . Equine Vet J 41: 82–86. 19301587 10.2746/042516408x330374

[COV019C43] Tang J Faustman C Mancini RA Seyfert M Hunt MC (2006) The effects of freeze–thaw and sonication on mitochondrial oxygen consumption, electron transport chain-linked metmyoglobin reduction, lipid oxidation, and oxymyoglobin oxidation . Meat Sci 74: 510–515. 22063055 10.1016/j.meatsci.2006.04.021

[COV019C44] Trumble SJ Noren SR Cornick LA Hawke TJ Kanatous SB (2010) Age-related differences in skeletal muscle lipid profiles of Weddell seals: clues to developmental changes . J Exp Biol 213: 1676–1684. 20435818 10.1242/jeb.040923

[COV019C45] Velten BP Dillman RM Kinsey ST McLellan WA Pabst DA (2013) Novel locomotor muscle design in extreme deep-diving whales . J Exp Biol 216: 1862–1871. 23393275 10.1242/jeb.081323

[COV019C46] Wagner A Marc A Engasser JM (1992) The use of lactate dehydrogenase (LDH) release kinetics for the evaluation of death and growth of mammalian cells in perfusion reactors . Biotechnol Bioeng 39: 320–326. 18600948 10.1002/bit.260390310

[COV019C47] Watson RR Miller TA Davis RW (2003) Immunohistochemical fiber typing of harbor seal skeletal muscle . J Exp Biol 206: 4105–4111. 14555750 10.1242/jeb.00652

[COV019C48] Watson RR Kanatous SB Cowan DF Wen JW Han VC Davis RW (2007) Volume density and distribution of mitochondria in harbor seal (*Phoca vitulina*) skeletal muscle . J Comp Physiol B 177: 89–98. 16924524 10.1007/s00360-006-0111-x

[COV019C49] Weise MJ Costa DP Kudela RM (2006) Movement and diving behavior of male California sea lion (*Zalophus californicus*) during anomalous oceanographic conditions of 2005 compared to those of 2004 . Geophys Res Lett 33: L22S10.

[COV019C50] Zhi W Srere PA Evans CT (1991) Conformational stability of pig citrate synthase and some active-site mutants . Biochemistry 30: 9281–9286. 1892835 10.1021/bi00102a021

